# Can a shift to regional and organic diets reduce greenhouse gas emissions from the food system? A case study from Qatar

**DOI:** 10.1186/s13021-020-00167-y

**Published:** 2021-01-09

**Authors:** José Luis Vicente-Vicente, Annette Piorr

**Affiliations:** Leibniz Centre for Agricultural Landscape Research (ZALF), Eberswalder Straße, 84, 15374 Müncheberg, Germany

**Keywords:** CO_2_ emissions, GHG emissions, C cycle, Organic production, Regional production, Agri-food system, Arid areas, Global food supply chains

## Abstract

**Background:**

Qatar is one of the countries with the highest carbon (C) footprints per capita in the world with an increasing population and food demand. Furthermore, the international blockade by some countries that is affecting Qatar—which has been traditionally a highly-dependent country on food imports—since 2017 has led the authorities to take the decision of increasing food self-sufficiency. In this study we have assessed the effect on greenhouse gas (GHG) emissions of shifting diets from conventional to organic products and from import-based diets to more regionalized diets for the first time in a Gulf country.

**Results:**

We found that considering the production system, the majority of the emissions come from the animal products, but the differences between conventional and organic diets are very small (738 and 722 kg CO_2_-eq capita^−1^ year^−1^, of total emissions, respectively). Conversely, total emissions from plant-based products consumption might be around one order of magnitude smaller, but the differences in the emissions between the organic and conventional systems were higher than those estimated for animal products, leading to a decrease in 44 kg CO_2_-eq capita^−1^ year^−1^ when changing from 100% conventional to 50% of organic consumption of plant-based products. Regarding the shift to regionalized diets, we found that packaging has a small influence on the total amount of GHG emissions, whereas emissions from transportation would be reduced in around 450 kg CO_2_ capita^−1^ year^−1^ when reducing imports from 100 to 50%.

**Conclusions:**

However, these results must be read carefully. Due to the extreme adverse pedoclimatic conditions of the country, commercial organic regional livestock would not be possible without emitting very high GHG emissions and just only some traditional livestock species may be farmed in a climate-friendly way. On the other hand, organic and regional low-CO_2_ emission systems of plant-based products would be possible by implementing innovations in irrigation or other innovations whose GHG emissions must be further studied in the future. Therefore, we conclude that shifting towards more plant-based organic regional consumption by using climate-friendly irrigation is a suitable solution to both increasing self-sufficiency and reducing C footprint. We encourage national authorities to including these outcomes into their environmental and food security policies.

## Background

Mitigating climate change by decreasing global greenhouse gas (GHG) emissions is currently one of the main challenges that science and society are facing. Food systems (FS), which include all processes and actors involved in the production, aggregation, processing, distribution, consumption and disposal of food products [1], are currently responsible for up to 37% of global GHG emissions [2], playing a key role in driving climate change [3]. Improving the sustainability of FS would require deep transformations comprising consumption patterns, system changes (e.g. management practices and distribution processes) and changes in the FS-environment interactions (e.g. governance).

Among all these processes involved in FS, shifting diets are one of the most important as climate change mitigation option. More plant-based, organic and regional-based diets have been proposed as a way to decrease GHG emissions [3–8]. In this line, the IPCC estimates—with medium confidence—that the total technical mitigation potential of dietary changes might be as 0.7–8 GtCO_2_-eq year^−1^ by 2050 [9].

There are different approaches and tools to estimate GHG emissions from the FS. Some of them include life cycle assessments (LCA), for specific crops, products or production systems [10–13]. However, when assessing the entire supply chain, LCA are not suitable and the lack of data becomes a problem. For instance, FAO estimations of GHG emission intensities for the different food products are calculated considering only “emissions generated within the farm gate”. Therefore, emissions from other upstream and downstream consumption and production processes are not included in the assessment [14]. An intermediate solution to address this fact is to consider many processes by using default data in order to create “calculators” or tools to estimate the environmental impacts of different production systems in specific places, assessing specific crop types or consumption patterns [15–19]. Even though these calculations might be not entirely precise [20, 21], they are considered suitable when comparing production systems (e.g. conventional vs organic) in order to assess policy measures, or to develop assessments combining CO_2_ emissions with economic tools (e.g. bioeconomic models) [22–25]. For instance, some high-quality studies assessing GHG emissions from fresh products applying LCA methodologies have been already published [11], but they lack on distinguishing between production systems, or do not consider downstream processes (i.e. transportation, refrigeration and packaging) that can be very relevant when assessing the FS at country level [26–29]. Addressing this research gap is especially relevant for those countries under highly specific pedoclimatic and/or socio-economic conditions, like Qatar, the focus of this study, where the extreme arid conditions—80 mm of annual precipitation and an evaporation rate of 2000 mm—have limited the agriculture to the production of some specific plant-based crops.

Indeed, the only suitable soils for conventional agriculture in Qatar are the “rodat” soils, those located in depressions and made up of calcareous loam, sandy loam and sandy clay loam with depths between 30 and 150 cm [30], where some natural vegetation grow [31]. However, these soils cover only a surface 28,000 ha [32] of a total of 67,000 ha of cultivable land [33]. The combination of this predomination of sandy soils and the huge gap in the water balance is now being compensated in agriculture with the extraction of high vulnerable groundwater resources [34, 35]. As an example, in 2012 the growndater extraction rate was about 400 Mm^3^ year^−1^, where between 236 and 250 Mm^3^ [34, 36, 37] were used for agriculture, whereas the natural replenishment rate is only about 60 Mm^3^ [34]. In order to address the depletion of the groundwater resources some authors have already proposed the use of environmentally-friendly measures, like the use of wastewater from agriculture or the deployment of low-CO_2_ emission technologies in the desalination process [36, 38].

In addition to these biophysical constraints for food production, Qatar is experiencing since mid-2017 a blockade by some neighbouring countries that has led to a significant increase in the costs of the imported food, mainly due to the increase in the complexity of logistics [39], and leading the country to establish specific targets and pathways to increase domestic self-sufficiency for the upcoming years [40]. However, this increase in the country´s self-sufficiency has to be coupled with the targets of the Paris Agreement [41], aimed at achieving net-zero CO_2_ emissions by 2050 and encouraging countries to accounting for the sources and sinks of the GHG emissions in order to calculate the Nationally Determined Contributions (NDCs), and therefore, including the emissions from the FS. This net-zero emissions target is very challenging for Qatar, since its carbon (C) footprint is among the highest in the world—around 44 t C capita^−1^ year^−1^—, and even much higher than some of its surrounding countries in the Gulf area [42].

In order to address the double challenge of Qatar of increasing local food self-sufficiency under extreme biophysical constraints while at the same time decreasing the C footprint the aim of this study is to: (1) develop a methodology to estimate GHG emissions based on the statistical available data and the official guidelines in order to compare alternatives for a FS transformation which are first, two management systems (conventional vs organic) and, second, the territorial scale of the supply chain (regional vs imports-based); (2) to apply the methodology to the conditions of Qatar; and (3) based in the results, to propose specific shifts in the diets in order to decrease GHG emissions while maintaining self-sufficiency goals.

In the following chapter we will introduce the delineation and methodology for the FS and scenario approach as well as data categories, sources and calculations applied for the entire chain assessment, followed by the description of the methodology to estimate the emissions related to the relevant steps of the supply chain. Chapter 3 presents and discusses the results with regard to a shift of production systems and transportation distances, as well as dietary shifts and contextualized the results beyond the system borders. The conclusions highlight the value of our findings and include policy recommendations.

## Methods

### Food systems and scenarios

**Fig. 1:**
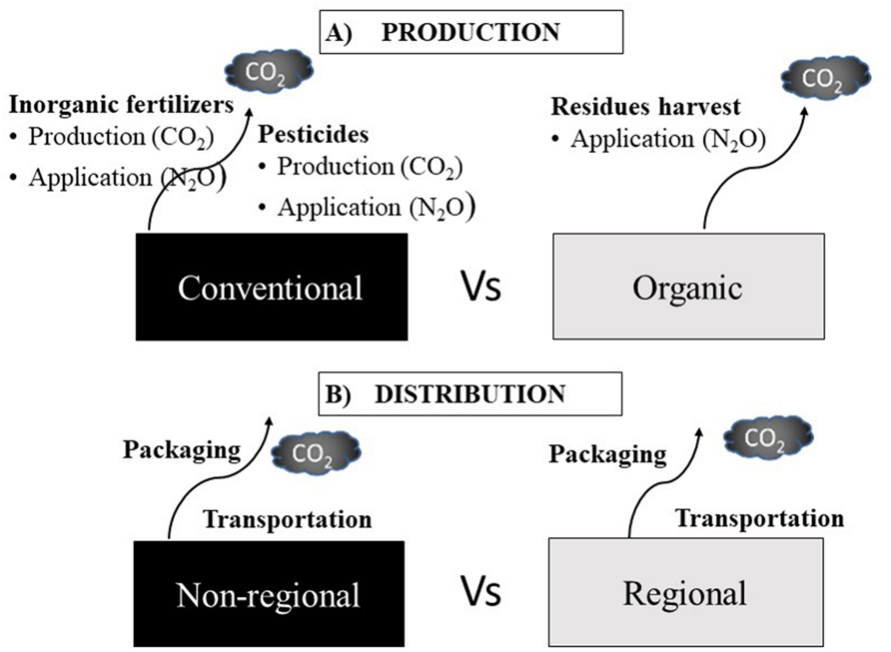
Scheme of the production (**a**) and distribution (**b**) assessments. Emissions from production of plant-based products are those resulting from the application of fertilizers and pesticides, whereas emissions from distribution are those from packaging and transportation. Emissions from the production of animal products are a result of life-cycle assessments from a literature review

Two different type of systems were selected for both, plant-based and animal products:

1. Management system (production): conventional vs organic. For plant-based products GHG emissions were specifically calculated for the different sources (Fig. 1a), whereas for animal products emission intensities for each system have been obtained from a literature review on life cycle assessments (LCA).
2. Territorial scale of the supply chain (distribution): regional vs non-regional (i.e. imports-based). Emissions from packaging and transportation of plant-based and animal products are included here (Fig. 1b).

Three type of scenarios were considered for the assessment:

1. Business as Usual (BAU). It defines the current state of the FS. When comparing the production systems BAU is considered to be 100% conventional. When comparing distribution systems, BAU has been calculated according to the imports of the country in 2015 (85% imports).
2. Complete adoption of one of the systems (100% conventional/organic and 100% regional/non-regional). Note that the 100% conventional is assumed to be the BAU for the production scenarios.
3. Half adoption of the system (50% conventional + 50% organic, and 50% regional + 50% non-regional).

Thus, for the assessment of GHG emissions from the production three scenarios have been assessed (100% conventional, 100% organic, and half adoption), whereas when assessing emissions linked to the distribution—packaging and transportation—four scenarios have been considered (BAU, 100% non-regional, 100% regional, and half adoption). The 100% organic and the 100% regional were considered as the baseline (i.e. net-zero emissions) scenarios when assessing differences in the GHG emissions between the different scenarios.

### Data management for plant-based products and literature review for animal products

**Fig. 2:**
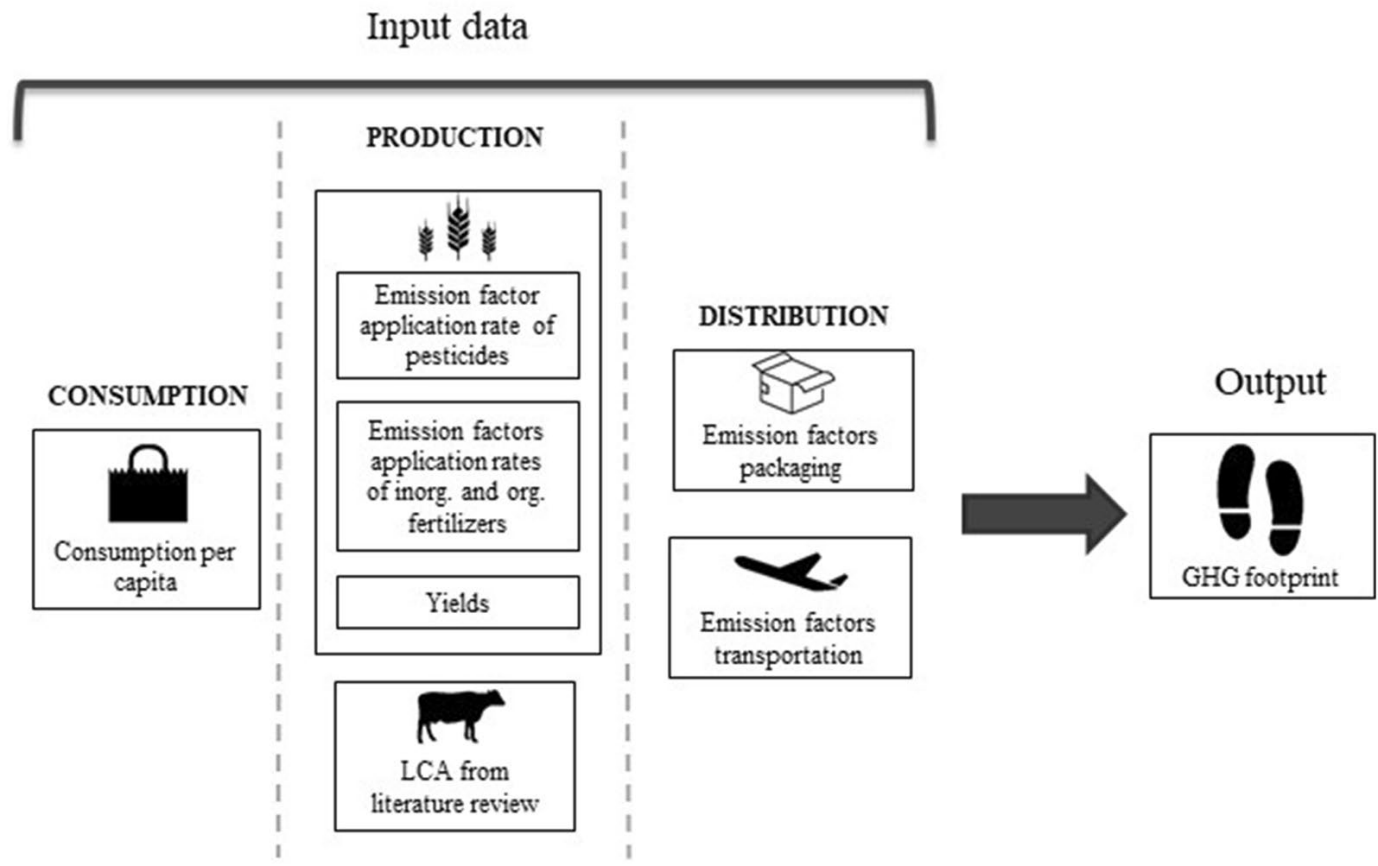
Scheme of the data requirements for the calculation of the GHG footprint. Data on food consumption, production (plant-based and animal products) and distribution (packaging and transportation) are needed to calculate the GHG footprint

Figure 2 shows a scheme of the data requirements for the estimation of the GHG footprint.

#### a. Consumption and yields

Data on food consumption are official publicly available from the State of Qatar [43] (Table 1). Some products have been excluded from the assessment due to the infeasibility of being produced in the country or lack of data on the production (nuts, tea, coffee, cacao, alcoholic drinks, pork, and oilseeds). The proportion of different consumed red-meat categories has been estimated by using FAO’s available data [44] from a similar country (United Arab Emirates, UAE). Cereals category includes wheat, barely, maize and other cereals. Vegetables category includes onions, beans, potatoes, sugar beet, tomatoes, cucumbers, cabbages, asparagus, carrots and turnips, cauliflowers and broccoli, pumpkins, eggplants, spinach, lettuce and chicory. Fish was assumed to be entirely farmed fish. Yields of plant-based products were taken from official statistics from the State of Qatar [45] (Table 1).

**Table 1:** Consumption (Kg capita^−1^ year^−1^) and yields (t ha^−1^ year^−1^) of the different plant-based and animal products included in the study

Product	Consumption	Yields
Eggs	11.69	
Poultry meat	43.41	
Fish (farmed fish)	16.05	
Milk (included dairy products)	90.61	
Beef cattle	8.39	
Sheep and goat meat	*13.50*	
Sub-total animal products	*183.65*	
Cereals	109.62	6.03
Vegetables	160.27	19.95
Fruits	43.45	3.90
Dates	14.56	12.00
Sub-total plant-based products	*327.90*	
Total animal and plant-based products	*511.55*	

Values taken from the official statistics of the State of Qatar [43, 45]

Values in italic show sub-total and total consumption

#### b. Production

##### b.1. Plant-based products

Pesticides and inorganic fertilization: Emission factor of pesticides is the value from Audsley et al. [46], a default value for every country. Specific application rates are taken from FAO [33] (Table 2). Emission factor of inorganic fertilizers (production, transportation, storage and transfer) is the one proposed for Asia by Kool et al. [47]. Specific application rates for Qatar are not available and, therefore, rates from UAE were taken [33] (Table 2).

**Table 2:** Emission factors and application rates of inorganic fertilizers in conventional agriculture

Pesticides	N-fertilizer	P_2_O_5_	K_2_O	
25.5	6.92	1.66	1.47	Emission factor (Kg CO_2_-eq Kg^−1^)
4.25	180.74	49.53	145.95	Application rate (Kg ha^−1^)

Organic fertilization: Due to the lack of data on the application of organic inputs in Qatar’s agricultural systems, in this study it was assumed that only residues from harvest were applied as organic inputs. Values of nitrogen (N) content in the residues were taken from Esteban et al*.* [48]. When N content was unknown, values from Williams et al*.* [49] were used. For the calculations of the amount of residues applied, it was assumed that all the residues from harvest would be applied and, for that purpose data on residue-to-product ratio were taken from different studies [50–54] (see Additional file 1: Table S1). Soil organic carbon (SOC) sequestration from the application of residues from harvest was not considered in the study due to the lack of data on decomposition rates and organic C dynamics in soils under extreme arid conditions.

Irrigation: Due to lack of data and high uncertainty on the current situation of crop managements, emissions from crop irrigation were excluded from the whole calculations. However, a specific section about it was included in order to obtain some first insights on the potential emissions from irrigation and, based on them, to propose specific environmentally-friendly alternatives (see “GHG emissions from the production” section).

##### b.2 Animal products

Data on emission intensities from livestock production in Qatar are not available [11]. Therefore, a literature review of emission intensities for conventional and organic systems was carried out. The products assessed were milk from cows, beef, poultry, pork, sheep and goat, and fish. Although pork is neither produced nor consumed in Qatar it was also included in the assessment in order to obtain a more complete review on factors to be possibly applied to other study cases in the future. For the literature review only those studies distinguishing between emissions from organic and conventional systems and assessing only the production process (i.e. transportation and packaging not included) were considered. A summary of the average emission intensities for each product is shown in Table 3. Complete data are shown in see Additional file 1: Tables S2–S9.

**Table 3:** Average emission intensities for animal products (Kg CO_2_-eq kg product^−1^)

Product	Conventional	Organic
Cow milk	1.06	1.06
Beef cattle	13.43	12.28
Poultry meat	4.6	6.7
Pork	3.92	2.99
Sheep and goat meat	17.5	10.10
Eggs	5.58	7.05
Farmed fish	1.77	0.87

Please, see Additional file 1: Table S2–S9 for detailed information on the literature review

#### c. Distribution

##### Transportation

**Fig. 3:**
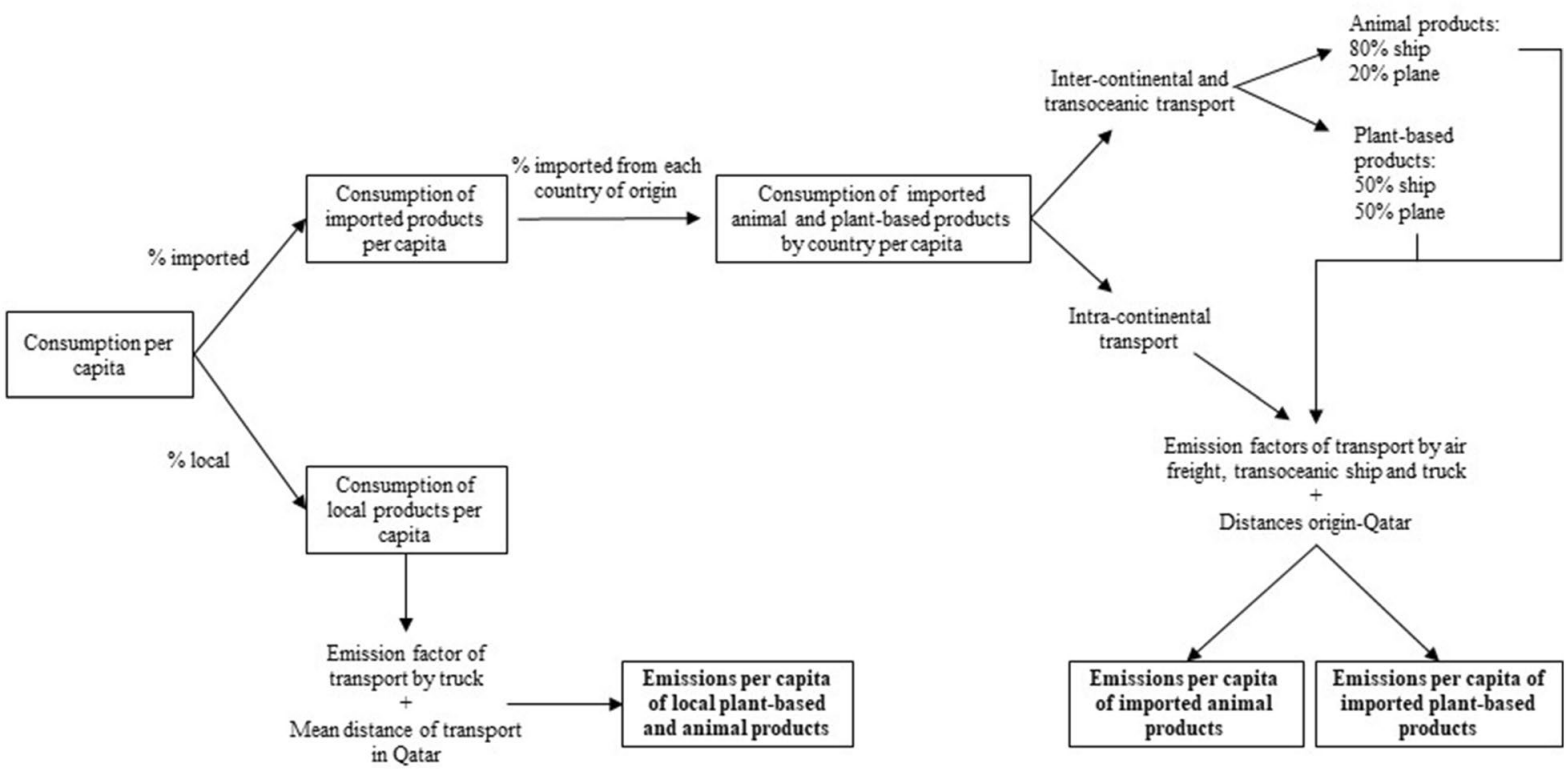
Scheme of the steps followed for calculating the emissions from transportation. % of local and imported food and emission factors of different means of transport are considered. For estimating emissions from imported food, the country of origin and type of product (animal or plant-based) are considered. After the application of the methodology three different types of GHG emissions are obtained: (1) from local plant-based and animal products, (2) from imported animal products and (3) from imported plant-based products

Figure 3 shows an overview of the methodology followed for the emissions from transportation. Since the data on imports is from 2013 [43] (see Additional file 1: Table S10), namely before the blockade of 2017, terrestrial transportation was assumed to take place by truck within Qatar (average capacity 7.5—16 tones) and between Qatar and the neighbor countries (average capacity 16—32 tones). For imports from further countries (see “Methodology to estimate CO_2_ emissions from transportation” section), ship or plane transportation were assumed. Emission factors for the different means of transport are shown in Table 4 [55, 56].

**Table 4:** Emission factors for the different means of transportation considered in the study. Adapted from Ecoinvent [55] and Heller [56]

	Kg CO_2_ Kg product^−1^ km^−1^
Truck, 7.5–16 metric tons	0.000302
Truck, 16–32 metric tons	0.000167
Transoceanic ship, freight	0.000022
Air freight	0.00112

Refrigeration is included in the estimations

##### Packaging

Emission factors from packaging were estimated considering values from different studies. They were grouped according to the type of commodity [57–60] (Table 5).

**Table 5:** Average emission factors from packaging of dairy products, meat, fish and vegetables and fruits

Product	Kg CO_2_ Kg product^−1^	References
Dairy products	0.053	Lindethal et al.[57], Williams and Wilkström [59]
Meat	0.150	Williams and Wilkström [59]
Fish	0.122	Ziegler et al. [60]
Vegetables and fruits	0.062	Lindethal et al*.* [59], Sonesson et al*.* [58]

### ***Methodology to estimate CO***_***2***_*** emissions from the production of plant-based products***

In the following sub-sections calculations of the emissions from the processes shown in the Fig. 1 are specified.

#### a. Conventional management

##### Pesticides

Emissions from the use of pesticides in conventional agriculture were calculated by using the Eq. 1.

1 $${\text{CO}}_{\text{2 pesticides}} = {\text{EF }} \times {\text{AR}}$$

where EF is the emission factor of the pesticide (production, transportation, storage and transfer) (Kg CO_2_ Kg pesticide^−1^) and AR the average pesticide application rate for Qatar (Kg pesticide ha^−1^).

##### Inorganic fertilization

Emissions from inorganic fertilization (Eq. 2) come from the production of the specific fertilizer (N, P or K-based) (Eq. 3) (Eq. 4) and the N_2_O emissions resulting from the application of the N-fertilizer (Eq. 5) (Kg CO_2_-eq ha^−1^) [61, 62]

2 $${\text{CO}}_{{\begin{array}{*{20}c} {2 - eq \; inorg. fert.} \\ \\ \end{array} }} = {\text{CO}}_{{2{\text{ N}},{\text{P}},{\text{K}} - {\text{fertilizers}}}} + {\text{CO}}_{2 - eq} [{\text{N}}_{2} {\text{O}}]_{N - {\text{fertilizer}}}$$

3 $${\text{CO}}_{{2{\text{ N}},{\text{P}},{\text{K}} - {\text{fertilizer}}}} = {\text{CO}}_{{2{\text{ N}} - {\text{fertilizer}}}} + {\text{CO}}_{{2{\text{ P}} - {\text{fertilizer}}}} + {\text{CO}}_{{2{\text{ K}} - {\text{fertilizer}}}}$$

where CO_2_ emissions for each fertilizer (Kg CO_2_ ha^−1^) are calculated as follows:

4 $${\text{CO}}_{{2{\text{ N}},{\text{P}},{\text{K}} - {\text{fertilizer}}}} = {\text{EF}}_{{{\text{N}},{\text{P}},{\text{K}} - {\text{fertilizer}}}} { } \times {\text{AR}}_{{{\text{N}},{\text{P}},{\text{K}} - {\text{fertilizer}}}}$$

where EF is the emission factor of the specific inorganic fertilizer (N, P, K) (production, transportation, storage and transfer) (Kg CO_2_ Kg fertilizer^−1^) and AR the application rate of each inorganic fertilizer (Kg fertilizer ha^−1^).

5 $${\text{CO}}_{2 - eq} [{\text{N}}_{2} {\text{O}}]_{{{\text{N}} - {\text{fertilizer}}}} = {\text{AR }} \times 0.01{ } \times { }{\raise0.7ex\hbox{${44}$} \!\mathord{\left/ {\vphantom {{44} {28}}}\right.\kern-\nulldelimiterspace} \!\lower0.7ex\hbox{${28}$}} \times 298$$

where AR is the application rate of the N-fertilizer (Kg fertilizer ha^−1^), 0.01 is the IPCC emission factor for added nitrogen, 44/28 is the conversion factor to transform to N_2_O emissions, and 298 is the global warming potential for nitrous oxide [61–63]

For both, CO_2-_eq from pesticides and inorganic fertilization, results are given per surface unit (hectares). In order to convert them to emissions per capita (Kg CO_2-_eq capita^−1^) the following equation was applied (Eq. 6):

6 $${\text{CO}}_{{2 - {\text{eq}}}} = {\text{CO}}_{{\begin{array}{*{20}c} {2 - eq \; inorg. fert.} \\ \\ \end{array} }} { } \times { }\frac{1}{Y} \times {\text{C}}$$

where $${\text{CO}}_{\begin{array}{c}2-eq \; inorg. fert.\\ \end{array}}$$ are the CO_2-_eq emissions per surface unit (Kg CO_2-_eq ha^−1^), Y is the yield for the specific product (Kg product ha^−1^) and C is the consumption per capita of the product (Kg product capita^−1^).

#### b. Organic management

Application of residues from harvest were considered as organic fertilizer. The calculation of the emissions (Kg CO_2_-eq Kg product^−1^) were done as follows (Eq. 7):

7 $${\text{CO}}_{2 - eq} [{\text{N}}_{2} {\text{O}}]_{org. fert.} = {\text{RP}} \times {\text{N }} \times 0.01{ } \times { }{\raise0.7ex\hbox{${44}$} \!\mathord{\left/ {\vphantom {{44} {28}}}\right.\kern-\nulldelimiterspace} \!\lower0.7ex\hbox{${28}$}} \times 298$$

where RP (Kg residue Kg product^−1^) is the residue-to-product ratio, N is the nitrogen content of the residue (Kg N Kg residue^−1^), 0.01 is the IPCC emission factor for added nitrogen, 44/28 is the conversion factor to transform to N_2_O emissions and 298 is the global warming potential for nitrous oxide.

### ***Methodology to estimate CO***_***2***_*** emissions from the production of animal products***

In this case, due to the lack of information on GHG emissions from livestock activities in arid areas [11] a literature review was carried out (see “Data management for plant-based products and literature review for animal products” section and Additional file 1: Tables S2–S9). In the literature review only those studies developing a LCA of the production phase were considered. GHG emissions from the consumption of animal products (Kg CO_2_-eq capita^−1^) were calculated as follows (Eq. 8):

8 $${\text{CO}}_{{2 - {\text{eq }}animal}} = {\text{C }} \times {\text{EF}}$$

where C is the consumption per capita of the specific product (Kg product capita^−1^) and EF is the emission factor (i.e. emission intensity) of the production of the animal product (Kg CO_2_-eq Kg product^−1^).

### ***Methodology to estimate CO***_***2***_*** emissions from transportation***

To calculate emissions from imports a similar methodology to that developed by Scholz [64] was followed (Fig. 3). First, the amount of plant-based and animal products of each country of origin was calculated (see Additional file 1: Table S10). The proportion of the imported product from each country was estimated by using available data on the amount of dollars spent on importing animal and plant-based products for 2015 [65]. For that assessment it was assumed that the proportion of dollars used for the imports is equal to the proportion of tons of imported food. This assumption was necessary due to the lack of information on the amount of food imports for each commodity. The next step is to consider the mean of transport. For plant-based products, it was assumed than all the intracontinental terrestrial transport comes by truck, whereas for intercontinental and transoceanic transport it was assumed that the 50% of plant-based and 80% of animal products come by ship, and the remaining come by plane. This assumption was based on the fact that most crop-based products are perishable and must be transported in just a few days [40, 64, 66].

Then, the CO_2_ emissions from the transportation of each product were calculated as follows (Kg CO_2_ capita^−1^) (Eq. 9):

9 $${\text{CO}}_{{2\;transportation}} = {\text{C }} \times {\text{EF }} \times {\text{D}}$$

where C is the consumption of the product per capita (Kg product capita^−1^), EF is the emission factor of the mean of transport (Kg CO_2_ Kg product^−1^ km^−1^) and D is the distance (Km) between the country of origin and Qatar.

For calculating the distances by plane Google Maps was used, whereas for calculating the distances by ship the website Sea Distances [67] was used. The most important port of the country was selected as port of origin. In case of different important ports existing in the country, the nearest port to Qatar was selected.

### ***Methodology to estimate CO***_***2***_*** emissions from packaging***

CO_2_ emissions from packaging are linked to the regionalization of the FS. In this study it was assumed that products coming from local production are not packaged, with the exception of milk, dairy products, eggs and cereals, which were considered to be packaged regardless of the place of production. Thus, CO_2_ emissions from packaging (Kg CO_2_ capita^−1^) were calculated as follows (Eq. 10):

10 $${\text{CO}}_{{2\;packaging}} = {\text{C }} \times {\text{EF}}$$

where C is the consumption of the product per capita (Kg product capita^−1^) and EF is the emission factor of the packaging of the specific type of product (Kg CO_2_ Kg product^−1^).

## Results and discussion

### GHG emissions from the production

#### a. Animal products

Emission intensities of meat from ruminants (beef, sheep and goat) account for the highest values (> 10 kg CO_2_-eq Kg product^−1^) mainly due to the enteric fermentation producing methane (CH_4_). Intermediate values are found for monogastric animal meat and eggs (3–7 kg CO_2_-eq Kg product^−1^) and the lowest values for milk from cows (1 kg CO_2_-eq Kg product^−1^) (Table 3). For monogastric—and also ruminants—emissions come from the CH_4_ releases from the stored manure, which also emits nitrous oxide (N_2_O) and in a lesser extent to the CO_2_ from the fossil fuels and energy usage [68].

The literature review indicates that the emissions per unit of product are similar between the two systems, conventional and organic, although there are differences between the specific products (Table 3). For instance, for milk from cows the same emission factor was found (1 kg CO_2_-eq Kg product^−1^) due to the similar values reported by the authors [49, 57, 69–74]. Similar findings occur with beef (12–14 kg CO_2_-eq Kg product^−1^) [73]. On the other hand, for fish (1.77 vs 0.87 kg CO_2_-eq kg product^−1^) [60, 75, 76], sheep (17.5 vs 10.1 kg CO_2_-eq Kg product^−1^) [49] and pork (3.9 vs 3.0 kg CO_2_-eq Kg product^−1^) [49, 73, 77] the conventional management has been reported to emit more CO_2_ than the organic. Conversely, the organic management account for higher emission intensities for poultry meat (6.7 vs 4.6 kg CO_2_-eq Kg product^−1^) [49, 73] and eggs (7.1 vs 5.6 kg CO_2_-eq Kg product^−1^) [49] (further information is shown in Additional file 1: Tables S2–S9).

For fish, more than 90% of the emissions came mainly from the feed. The reduction in the emissions in the organic system to almost half of the conventional is due to the change in the feed formulations (e.g. from fish and animal protein meals to vegetable-based meals) [75] (see Additional file 1: Tables S7 and S8). On the other hand, poultry consumes high value feeds and the nutritional needs are met by arable crops, whereas ruminants are able to digest cellulose and, therefore, can be fed by grass [49]. To produce arable crops in the conventional system, synthetic fertilizers are used and, therefore, more energy (i.e. more CO_2_ emissions) is required than in the organic system. However, due to the lower organic bird performance (i.e. lower efficient system because of the higher feeding requirements to produce the same amount of meat) the benefits of this lower energy requirements are over-ridden [49] (see Additional file 1: Table S4).

**Fig. 4:**
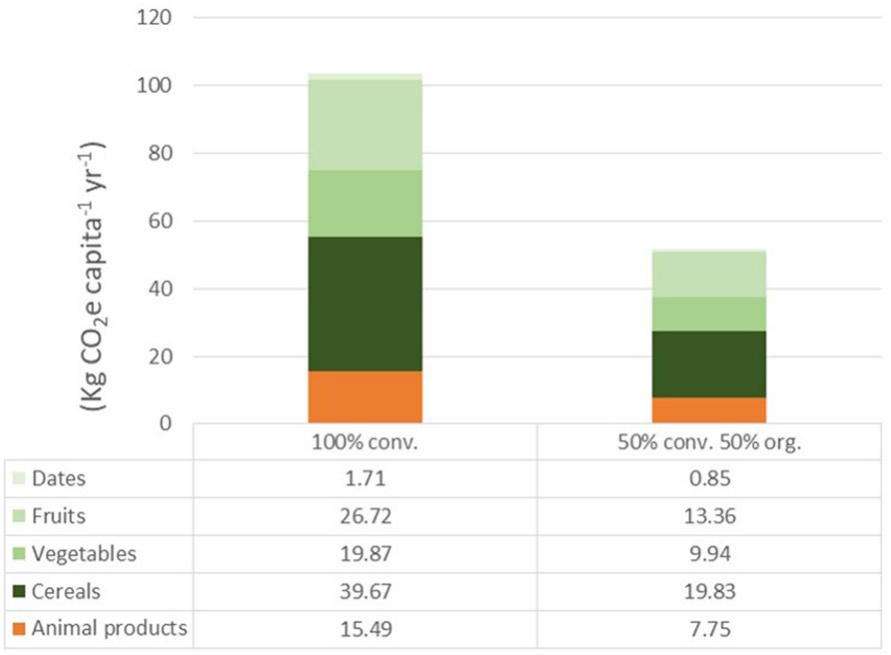
Differences in the CO_2_-eq emissions from the production of plant-based and animal products in the 100%-conventional, and for the 50%-conventional and 50%-organic scenarios. Differences in the emissions from the different plant-based commodities are shown. Note that the 100% conventional is the business-as-usual scenario, whereas the baseline (i.e. zero emissions) is the 100% organic

Regarding the differences in the CO_2_ emissions between conventional and the organic systems and considering the organic system as baseline (i.e. zero emissions), the emissions per capita from the conventional system—taken as the BAU scenario—amount 103 kg CO_2_-eq year^−1^, where around 85% of the reductions (88 kg CO_2_-eq capita^−1^ year^−1^) come from the plant-based products and 15% (15 kg CO_2_-eq capita^−1^ year^−1^) from the animal products (Table 6 and Fig. 4). This was due to the fact that, in average, average emission intensities of animal products in organic and conventional systems are very similar (6.34 and 7.32 kg CO_2_-eq Kg product^−1^, respectively) (Table 3), and so are the differences in the CO_2_ emissions.

**Table 6:** Total emissions and difference in the emissions from comparing conventional and organic production systems in the three scenarios

Food products	Consumption (Kg capita^−1^ year^−1^)	Emission intensities of animal products (Kg CO_2_ Kg product^−1^)		Emission intensities of plant-based products (Kg CO_2_ Kg product^−1^)		Scenario 1 (KgCO_2_-eq capita^−1^ year^−1^)	Scenario 2 (KgCO_2_-eq capita^−1^ year^−1^)	Scenario 3 (KgCO_2_-eq capita^−1^ year^−1^)
		Conventional	Organic	Conventional	Organic	100% conventional	100% organic	50% conv. 50% org
Eggs	11.69	5.58	7.05			65.25	82.44	73.84
Poultry meat	43.41	4.6	6.7			199.67	290.82	245.24
Pork meat								
Farmed fish	16.05	1.77	0.87			28.43	14.01	21.22
Milk and dairy products	90.61	1.06	1.06			95.90	96.04	95.97
Beef cattle	8.39	13.43	12.28			112.65	102.98	107.81
Sheep and goat meat	13.50	17.5	10.1			236.24	136.35	186.29
Total emissions of animal products						738.13	722.63	730.38
Difference in the emissions						*15.49*	*0*	*7.75*
Cereals	109.62			0.42	0.05345	45.53	5.86	25.69
Vegetables	160.27			0.13	0.00140	20.10	0.23	10.16
Fruits	43.45			0.64	0.02661	27.88	1.16	14.52
Dates	14.56			0.21	0.09132	3.04	1.33	2.18
Total emissions of plant-based products						96.54	8.57	52.55
Difference in the emissions						*87.97*	*0*	*43.98*
Total emissions (plant-based + animal products)						834.66	731.21	782.93
Total difference in the emissions (plant-based + animal products)						*103.46*	*0*	*51.73*

Separated results for animal and plant-based products in the three scenarios are shown. Please, note that the Scenario 1 is the business-as-usual (BAU) scenario and that the 100% organic (scenario 2) is the baseline for the calculation of the difference in the emissions

Values in italic show differences in the emissions

**Fig. 5:**
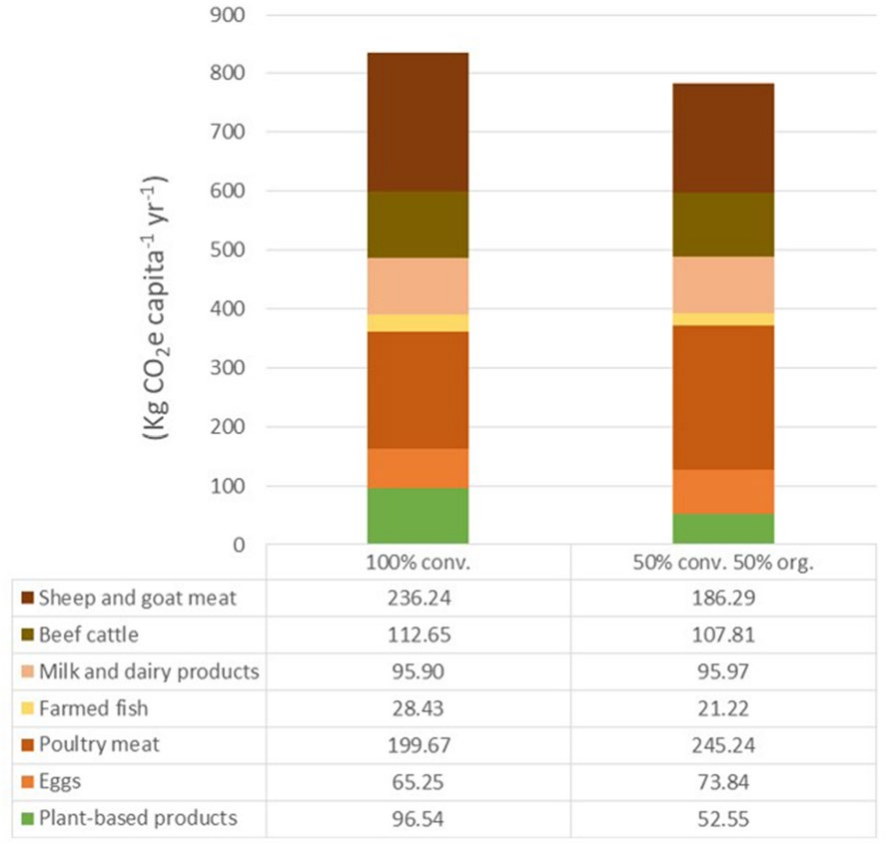
Total estimated CO_2_-eq emissions from the production of plant-based and animal products in the 100% conventional, and in the 50% conventional and 50% organic scenarios. Emissions from animal products are shown for each food commodity assessed in the study. Please, note that the emissions from plant-based products do not include some management activities that are common for both systems (e.g. tillage or harvesting)

However, in absolute numbers, the consumption of animal products amount the highest total CO_2_-eq emissions (738 and 730 KgCO_2_-eq capita^−1^, for the conventional and the 50% organic systems, respectively) (Table 6 and Fig. 5). This figure is similar to the 765 KgCO_2_ capita^−1^ value found in another study for United Arab Emirates [78]. This fact is explained because the emission intensities of animal products are much higher than those of the plant-based, leading to a much lower total CO_2_ emissions from the latter (9 and 97 KgCO_2_-eq capita^−1^, for the organic and the conventional systems, respectively) (Table 6 and Fig. 5). Therefore, we found that around 87% of the total GHG emissions come from animal products in the conventional system, whereas this value is even higher, around 98%, in the organic system. However, there were differences in the contribution of each animal-product commodity (Fig. 5). In the conventional system sheep and goat products accounted for the highest total emissions (236 KgCO_2_-eq capita^−1^), followed by poultry meat and beef (200 and 113 KgCO_2_-eq capita^−1^). In the 50% organic scenario the commodity accounting for the highest emissions was poultry meat (245 KgCO_2_-eq capita^−1^), followed by sheep and goat products and beef (186 and 108 KgCO_2_-eq capita^−1^). Milk and dairy products, and farmed fish accounted for lower values due to the much lower emission intensities, whereas emissions from eggs were lower due to the relatively low level of consumption and intermediate emission intensity values (Tables 1 and 3).

#### b. Plant-based products

**Fig. 6:**
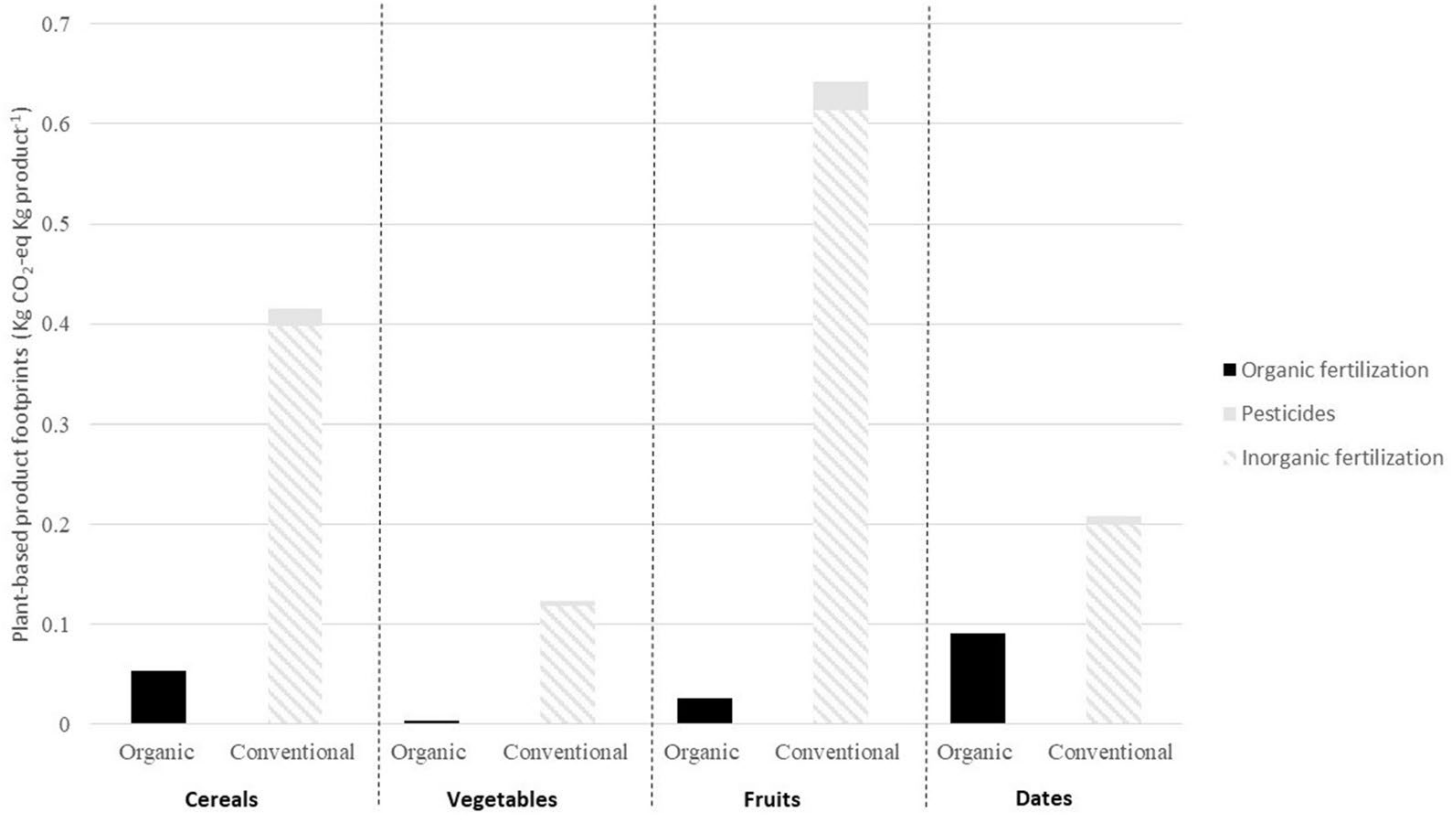
Plant-based CO_2_-eq emissions from the processes assessed in the production of plant-based products in organic and conventional systems: organic fertilization (application of residues from harvest), inorganic fertilization (production and application) and pesticides (production). Please, note that due to the lack of data the results shown on the graphic do not consider some potential sinks in the organic system (i.e. soil organic carbon sequestration from the application of residues from harvest and other organic inputs) as well as some potential emissions (i.e. N_2_O emissions from the application of other organic fertilizers)

For plant-based products GHG emissions are markedly higher in the conventional system. The use of inorganic fertilizers plays a key role on the total amount, accounting for more than 95% of the total emissions (Fig. 6). Emissions from inorganic fertilization come from two sources. First, the GHG emitted in the processes before being applied (production, transportation, storage and transfer) [47], and second the N_2_O emissions after their application [61, 62]. In average, around two thirds of the emissions belong to the fertilizer production (1547 kg CO_2_ ha^−1^), whereas the other third comes from the emissions that occur in the context of application (846 kg CO_2_-eq ha^−1^) [33, 61, 62]. Our results are in line to those shown in the official statistics of FAO, estimating around 0.20 kg CO_2_-eq Kg cereal^−1^ (world average) [33], whereas in our study the value for conventionally-produced cereals was slightly higher (0.42 kg CO_2_-eq Kg cereal^−1^) (Table 6). Emission intensities for vegetables accounted for the lowest emission value (0.13 kg CO_2_-eq ha^−1^), whereas this figure was of 0.21 and 0.64 kg CO_2_-eq ha^−1^ for dates and fruits, respectively. Thus, when considering the different level of consumption, a shift to organic production of cereals and fruits accounted for the highest GHG emission reductions (Table 6 and Fig. 4).

#### c. Irrigation

However, neither in our study nor in other similar studies (e.g.[33]) additional emissions from irrigation (i.e. water desalination) are included. This is due to the lack of accessible and accurate data on water requirements and current sources of water used for irrigating crops in Qatar—or countries under similar pedoclimatic conditions—and emission factors from water desalination. Nevertheless, in order to show an example of how irrigation from desalted water would imply in terms of CO_2_ emissions, we can give a rough estimation of the emissions from irrigation with desalted water for cereal production in Qatar. Thus, considering an estimated emission factor of 2.04 kg CO_2_ m^−3^ freshwater for desalted water (average value from different desalination processes from Liu et al*.* [79]) and an estimated required irrigation for cereals in Qatar of 1.52 m^3^ Kg cereal^−1^,^1^ around additional 3.1 KgCO_2_ would be emitted per kilogram of cereal produced. Furthermore, considering the feed requirements of beef, and poultry meat^2^ [81], 21.7 and 6.2 kg CO_2_ per kilogram of beef and poultry from desalted water would be emitted, respectively, if they were produced in Qatar.

Currently agriculture in Qatar relies mainly on groundwater sources [82], extracting around 250 Mm^3^ per year, when the sustainable rate would be around only 60 Mm^3^ [34, 37], and leading to an impoverishment of the groundwater quality (e.g. increase in the salinity) [82, 83]. Therefore, additional regional production should be based on the use of non-groundwater sources (i.e. at this moment desalted seawater) [36].

Although in this study CO_2_ emissions from irrigation are not included in the calculations of the production of regional plant-based products and neither the emissions from feeding the livestock, these estimations could give an idea about the order of magnitude of the additional emissions that regional production would imply. For regional plant-based products, additional emissions from irrigation would be around one order of magnitude higher than those from the production, whereas for animal-based products these could be in the same order of magnitude. However, the energy losses due to the decrease in the energy use efficiency from feeding animals lead, in absolute numbers (i.e. emissions per kilogram of final product), to higher emissions from irrigation from animal products.

Nevertheless, there could be lower-emission options to obtain water for irrigation. Among these options the use of wastewater—including the use of by-products from wastewater—has become one of the most important alternative water sources in the recent years [36, 84–86]. Furthermore, some lower-emission technologies could be applied to the desalination plants like hybrid systems (e.g. solar photovoltaic cells with wind energy, nanofiltration and ultrafiltration for pre-treatment, electrodialysis and reverse osmosis, forward osmosis with nanofiltration) [38, 87] or even some future CCU (Carbon Capture and Utilization) technologies to re-use the CO_2_ with the brine produced in the plant to produce carbonates after a mineralization process [88–92]. Recently, in this line, Namany et al. [93] using a holistic energy, water, and food (EWF) nexus approach in Qatar, found that diversifying the energy and water mix by introducing more than 70% of renewable energy technologies and utilizing reverse osmosis would decrease the environmental impact of this process by 60% from these two sectors.

### GHG emissions from the distribution (packaging and transportation)

**Fig. 7:**
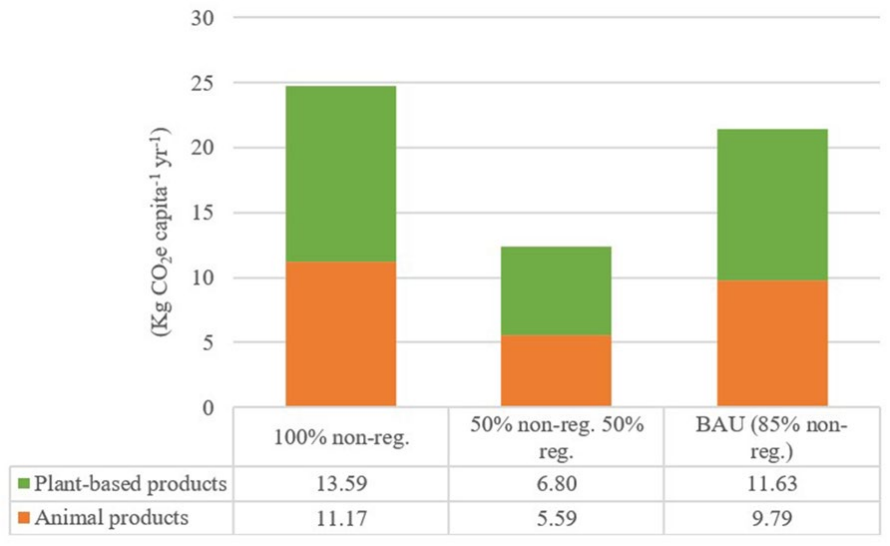
Differences in the CO_2_ emissions from the packaging in the distribution of plant-based and animal products in the 100%-non-regional, 50%-non-regional and 50%-regional, and business-as-usual (85% non-regional) scenarios. Please, note that the 100% regional is the baseline scenario (i.e. zero emissions)

Additional emissions from packaging in the 50%-regional scenario are only of 12 kg CO_2_ capita^−1^ higher than in the ideal case of 100% regional, whereas for the 100% non-regional and the BAU scenario (85% regional) these values are of 25 and 21 kg CO_2_ capita^−1^, respectively (Table 7 and Fig. 7). These values are four times lower than the additional emissions in the conventional system for the production of plant-based products, but in the range of the differences in the emissions in the production of conventional and organic animal products. Despite the emission factors of packaging of animal products are almost double of the plant-based, total emissions from plant-based products are slightly higher due to the higher consumption of the latter (Tables 1 and 5, and Fig. 7).

**Table 7:** Total emissions and difference in the emissions from packaging from comparing regional and non-regional distribution systems in the four scenarios

Food products	Consumption (Kg capita^−1^ year^−1^)	Emission intensities of animal products (Kg CO_2_ Kg product^−1^)		Emission intensities of plant-based products (Kg CO_2_ Kg product^−1^)		Scenario 1 (KgCO_2_ capita^−1^ year^−1^)	Scenario 2 (KgCO_2_ capita^−1^ year^−1^)	Scenario 3 (KgCO_2_ capita^−1^ year^−1^)	BAU (KgCO_2_ capita^−1^ year^−1^)
		Non-regional	Regional	Non-regional	Regional	100% non-regional (100% packaging)	100% regional (no packaging)	50% non-reg 50% reg	85% non-reg
Eggs	11.69	–	–			–	–	–	–
Poultry meat	43.41	0.15	0			6.51	0	3.26	6.01
Farmed fish	16.05	0.12	0			1.95	0	0.98	1.35
Milk and dairy products	90.61	–	–			–	–	–	–
Beef cattle	8.39	0.15	0			1.26	0	0.63	1.19
Sheep and goat milk meat	13.50	0.11	0			1.45	0	0.73	1.30
Difference in the emissions of animal products						*11.17*	*0.00*	*5.59*	*9.79*
Cereals	109.62			–	–	–	–	–	–
Vegetables	160.27			0.062	0	9.98	0	4.99	8.86
Fruits	43.45			0.062	0	2.71	0	1.35	2.66
Dates	14.56			0.062	0	0.91	0	0.45	0.10
Difference in the emissions of plant-based products						*13.59*	*0.00*	*6.80*	*11.63*
Total difference in the emissions (plant-based + animal products)						*24.76*	*0.00*	*12.38*	*21.41*

Separated results for animal and plant-based products in the four scenarios are shown. Please, note that the 100% regional (scenario 2) is the baseline for the calculation of the difference in the emissions

Values in italic show differences in the emissions

**Fig. 8:**
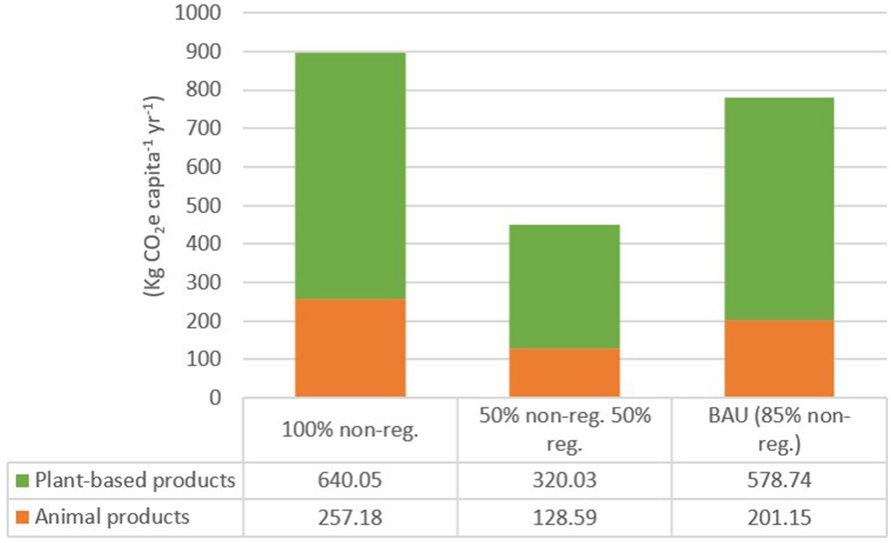
Differences in the CO_2_ emissions from the transportation in the distribution of plant-based and animal products in the 100%-non-regional, 50%-non-regional and 50%-regional, and business-as-usual (85% non-regional) scenarios. Please, note that the 100% regional is the baseline scenario (i.e. zero emissions)

Regarding the transportation, the additional emissions from the 50%-regional scenario compared to the 100% regional would be of around 450 kg CO_2_ capita^−1^, whereas for the 100%-non-regional and the BAU scenarios they are of 915 and 780 kg CO_2_ capita^−1^, respectively (Table 8 and Fig. 8). That means that if shifting from the BAU to the 50%-regional scenario, the saved emissions per capita would be around 330 kg CO_2_. That is one order of magnitude higher than the differences between systems found in in the production and packaging. Almost two thirds of the emissions belong to the plant-based products, whereas the other third comes from the transportation of the animal products (Table 8 and Fig. 8). This is due to two facts, (i) the higher consumption of plant-based products, and (ii) the higher proportion of perishable products (i.e. short shelf life and easily deterioration) in the plant-based products group that must be transported by plane [40, 66]. In this line, average emissions from transportation of regional products amounted 0.023 KgCO_2_ Kg product^−1^ for both plant-based and animal products, whereas for imported products emissions are remarkably higher, between 60 and 90 times for plant-based and animal products (1.47 and 1.92 kg CO_2_ kg product^−1^), respectively (Table 8) (see also Additional file 1: Tables S11–S13). These values are very similar to those calculated in a similar study in Sweden [64], where the average emissions per unit of imported food product were of 1.64 kg CO_2_.

**Table 8:** Total emissions and difference in the emissions from transportation from comparing regional and non-regional distribution systems in the four scenarios

	Consumption (Kg capita^−1^ year^−1^)		Emission factors of animal products (Kg CO_2_ Kg product^−1^)		Emission factors of plant-based products (Kg CO_2_ Kg product^−1^)		Scenario 1 (KgCO_2_ capita^−1^ year^−1^)	Scenario 2 (KgCO_2_ capita^−1^ yr^−1^)	Scenario 3 (KgCO_2_ capita^−1^ year^−1^)	BAU (KgCO_2_ capita^−1^ year^−1^)
	Animal	Plant-based	Non-regional	Regional	Non-regional	Regional Regional	100% non-regional	100% regional100% regional	50% non-reg 50% reg	85% non-reg
	183.65	327.90	1.4232	0.0227	1.9746	0.0227				
Total emissions of animal products							261.34	4.16	132.75	205.31
Difference in the emissions of animal products							*257.18*	*0*	*128.59*	*201.15*
Total emissions of plant-based products							647.48	7.43	327.45	586.17
Difference in the emissions of plant-based products							*640.05*	*0*	*320.03*	*578.74*
Total emissions (plant-based + animal products)							908.82	11.59	460.20	791.48
Total difference in the emissions (plant-based + animal products)							*897.23*	*0*	*448.62*	*779.89*

Separated results for animal and plant-based products in the four scenarios are shown. Please, note that the 100% regional (scenario 2) is the baseline for the calculation of the difference in the emissions. More information on imports and intermediate calculations are shown in Additional file 1: Tables S10–S13)

Values in italic show differences in the emissions

In Qatar the majority of the fodder used to feed animals in livestock is imported [40] and part of it comes from the US [94]. Considering the specific emission factor (Table 4) and distance by ship, around 0.33 KgCO_2_ per kilogram of transported product would be emitted. That means, for feeding regional livestock in Qatar emissions from the transportation of feed would be around 2.31 and 0.66 kg CO_2_ kg^−1^ for beef and poultry meat produced in Qatar, respectively. However, if the meat was not produced in Qatar but directly in the US and then imported to Qatar the emissions from transportation would be reduced to 0.33 kg CO_2_ kg product^−1^, in the case that they were transported by ship.

However, fodder production in Qatar could be increased and CO_2_ emissions from irrigation decreased by implementing Treated Sewage Effluent (TSE) facilities. In this line, Qatar has increased green fodder cultivated areas by combining groundwater and TSE more than three times in 11 years (2001–2012) reaching 5183 ha, whereas the area irrigated with only TSE sources was around 1520 ha in the year 2012 [86]. This increase in the use of TSE technologies would decrease the emissions associated to the regional food production, making the imports less sustainable in terms of GHG emissions and preserving groundwater sources [40, 86].

### ***Decreasing the CO***_***2***_*** footprint by shifting diets in Qatar***

#### a. Animal products

Due to the water scarcity, very high insolation and poor soils that characterize arid areas only very specific products can be produced regionally in a traditional way (e.g. dates [95] or camels [96]), although in the recent years new organic farms practicing greenhouse production have appeared. In general, organic livestock farming is carried out extensively, based on grazing (permanent grasslands, natural pastures, specific rotations…) and, therefore, in Qatar only conventional livestock farming can be implemented, as it can be carried out indoors by maintaining specific climatic conditions and by feeding partially the livestock with imports [97]. However, this leads to a high increase in the CO_2_ footprint compared to the animal products produced in temperate areas. In this sense, it is very important to highlight that, due to the lack of studies and data, the emission intensities from livestock come from LCA estimations from temperate areas and, therefore, they do not take into account the specificities of the livestock farming in Qatar (e.g. extra water and energy consumption) [97]. For example, according to our results (Tables 6 and 7) the consumption of 1 kg of imported conventional beef in Qatar would imply the emission of 13.87 kg CO_2_ (13.43, 0.15 and 0.29 kg CO_2_-eq kg product^−1^ from production, packaging and transportation, respectively) (97% from production) if it is frozen meat transported from Australia by ship, or 28.48 kg CO_2_ (13.43, 0.15 and 14.90 kg CO_2_-eq kg product^−1^ from production, packaging and transportation, respectively) (47% from production) in case it is fresh meat imported from Australia by plane. These results suggest that even though the additional emissions from commercial livestock farming in Qatar are not known, emissions from transportation and packaging would be negligible compared to those from the production in the total balance when transporting by ship (i.e. frozen meat or fresh meat from nearby countries).

An exception would be the traditional regional livestock. Traditional livestock species (e.g. camels, goats) which are used to the extreme conditions of Qatar could be fed by indigenous palatable plants and palatable halophytes, which consume less freshwater and, therefore, could be used as fodder or “pastures” [31]. These species could substitute the current exotic plants used for feeding the livestock [e.g. rhode-grass (*Chloris gavana*) and alfalfa (*Medicago sativa*)], which can consume up to 48,000 m^3^ ha^−1^ year^−1^ of water [96].

However, what is clear from our results is that decreasing the level of meat from ruminants could be an effective strategy to decrease the GHG emissions. Emissions from transportation and packaging remain similar but emissions from conventional production are three times lower for poultry meat, meaning a decrease in 8.83 kg CO_2_-eq Kg product^−1^ (from 13.43 to 4.6 kg CO_2_-eq kg product^−1^). Considering the actual consumption of beef in Qatar, the shift from beef to poultry meat would lead to a decrease of about 74 kg CO_2_-eq per capita and year. This relatively high reduction in the GHG emissions from the shift from ruminants to monogastrics is already very well-known and, therefore, in line with other studies showing differences between three times [98] until one order of magnitude [99], and suggesting that up to 65% of the world´s GHG from livestock would come from cattle [100]. Importantly, in terms of GHG emissions, the differences between the organic and conventional systems for the overall animal products are negligible.

#### b. Plant-based products

In our scenarios, we selected organic fruit and horticulture farming also due to the fact that they use organic inputs (residues from harvest, manure, pruning debris, sewage sludge, compost…) as fertilizers. However, they do not necessarily require soil, but they can be grown by using soil-free substrates) and water (i.e. soilless food production) or can be combined with aquaculture (i.e. aquaponics) [101]. In our study, emissions from the use of inorganic fertilizers and pesticides in the production of conventional vegetables averaged 0.12 kg CO_2_-eq Kg product^−1^ (Fig. 6). Considering an emission factor from packaging of 0.06 kg CO_2_ Kg product^−1^ (Table 5) and same emission factor for transportation than in the previous example for meat (Table 4), emissions from packaging and transportation would be higher than those from the production. Moreover, since the emissions from inorganic fertilizers and pesticides are relatively high compared to those from the organic system (Fig. 6), and considering the emissions from transportation and packaging, regional and organic farming might be considered as mitigating options.

Another mitigating option also suggested by many authors since the last decade[100] is decreasing the level of meat and dairy consumption and, thus, increasing plant-based products consumption (i.e. adopting vegetarian or vegan diets). Despite main focus of this study is not assessing the shift from animal to plant-based products, according to our results, reducing the consumption of animal products to half of the current level and substituting them with plant-based products would save around 368 KgCO_2_ capita^−1^.^3^ In this line, Joyce et al*.* [103] in a literature review found that shifting to non-meat diets could save up to half of the total diet-associated emissions compared to an average diet.

### Gaps, future researches, and synergies and trade-offs with other ecosystem services

#### a. Lack of studies in Qatar and countries under similar pedoclimatic conditions

The complete lack of studies in Qatar assessing emission factors and intensities in agriculture and livestock [11] led us to consider many assumptions. Emission intensities of animal products are taken from studies carried out in temperate areas, with very different conditions than those existing in Qatar, where water and energy requirements are typically much higher. For agriculture, UAE’s application rate of inorganic fertilization has been taken [33], whereas the emission factor from its production was taken from Asia’s default value from Kool et al. [47]. Similarly, the application rate of pesticides was taken from FAO Statistics for Qatar [33] and the emission factor from the production of pesticides was a default value from Audsley et al. [46].

Furthermore, due to the lack of studies on organic farming in arid areas SOC sequestration has not been considered in the study. Vicente-Vicente et al. [104] found in a meta-analysis in Mediterranean woody crops that the application of organic amendments could sequester up to 5 t C ha^−1^ year^−1^ (18 t CO_2_ ha^−1^ year^−1^). Our study has considered only as fertilizer the application of residues from harvest in the organic farming, thus excluding the application of other organic amendments (compost, manure, sewage sludge…), since their type, application rate and N dynamics depend highly on the specific local conditions (e.g. nearby livestock farms, nearby industries generating organic byproducts…) [104–107] and these data are not available in Qatar. Therefore, the reduction in the emissions in organic agriculture compare to the conventional system shown in this assessment must be taken as estimations since eventually depend on the balance between the N_2_O emissions and SOC sequestration after the application of the organic inputs. In this line, future studies assessing this balance under extreme arid conditions must be carried out.

#### b. System boundaries

The assessment, especially for the plant-based products, has been developed in order to compare systems (organic vs conventional and regional vs non-regional) (i.e. 100% organic and 100% regional have been considered as zero-emissions scenarios for the comparisons). When comparing organic vs conventional agricultural systems, only those practices that are different between the two systems have been considered. However, when comparing regional vs non-regional products, local specificities were not considered due to the many different origins of the imports and the complexity of the systems in each country. The result is that the potential extra CO_2_ emissions from irrigation in Qatar were not included when calculating emissions from regional products. In the same way, for livestock regionally produced in Qatar, additional emissions from importing the fodder or those from maintaining the climatic conditions in indoor facilities were not taken into account. Nevertheless, as we are aware of those processes, specific sections and estimations have been included in the study in order to figure out the order of magnitude of them (e.g. “GHG emissions from the production” section). However, we want to emphasize that future studies assessing the GHG emissions from the new and highly intensive livestock farms in Qatar are needed in order to develop more accurate calculations.

#### c. Synergies and trade-offs with other ecosystem services

Assessing the effect of different FS in terms of GHG emissions means assessing only one regulating ecosystem service [108]. However, fostering one specific FS also affects other ecosystem services beyond GHG emissions. For instance, organic farming improves soil supporting services, like SOC content [109] and, thus, it affects positively some soil fertility properties (e.g. microbial activity, soil porosity and water retention). Furthermore, organic farming might affect positively other regulating ecosystem services (e.g. pollination, biological control, biodiversity), whereas there might be some trade-offs especially with provisioning ecosystem services (e.g. food production) [110, 111]. On the other hand, traditional regional production fosters cultural and aesthetic ecosystem services like local economy, traditions and quality of the landscape [112–114]

However, fostering intensive commercial regional livestock (e.g. cows) increases country´s food production, but they might emit more GHG than the imported meat or milk because the climatic conditions of the country do not allow low-CO_2_ emissions intensive livestock farming. As a matter of fact, the great majority of the fodder in Qatar is not produced in the country but in far-distant countries “including the USA and other northern and southern hemisphere countries” [94], thus consuming land and resources in other countries and emitting extra GHG emissions. Therefore, new frames considering externalities beyond the country borders, like telecoupling [115], should be considered when assessing the impacts of a FS on ecosystem services, as the current FS cannot be isolated within the country, but they depend on international food chains. Thus, we found a clear trade-off between increasing country’s food production of non-traditional animal products and GHG emissions in Qatar. This trade-off could be mitigated through the increase in the production of traditional animal products (e.g. camels, goats, sheep) that can be fed with local plant species in an extensive way [96].

## Conclusions and recommendations for policymakers

In our study the ambition was to introduce and apply a methodology for a databased assessment of the potential for GHG emission savings associated with the transformation of the food system towards a more sustainable (organic) production system or a distance-related shortening of supply chains. With the emerging experiences regarding food chain resilience along the COVID-19 crisis the regionalization of global food chains became a broadly considered issue. In this course also the transformation towards more climate neutral and sustainable systems is addressed. Our study presents first assessments of a possible transformation scenarios resulting from a post-crisis situation, following the embargo situation in Qatar. We have purposely adapted our approach to the particularly conditions (e.g. pedoclimatic) of this country. Although the results and conclusions are to be valued specifically under these conditions, our methodological approach should also be useful for other case studies.

Achieving a complete GHG-neutral food system is not feasible, since every activity has an impact on GHG emissions and food consumption cannot be avoided. Even the SOC sequestration, which is the main sink of CO_2_ in the food system has a limit and is reversible. Therefore, comparing food production systems and commodities in terms of GHG emissions could be a suitable methodology when assessing the suitability of the different systems in the decision-making processes. In this regard, we found that for animal products, the majority of the emissions come from the production, regardless of the production system (organic or conventional), with the exception of the products coming by air freight, where transportation could contribute up to half of the total emissions. Due to the climatic conditions in Qatar, which make production of animal products more costly in terms of energy and water consumption than in other climates, imports by ship or truck would emit less GHG than regional production. Therefore, regional and conventional livestock farming of non-traditional animal species in Qatar would might not be beneficial in terms of overall GHG emissions. However, trade-offs between the local production of non-traditional animal products and GHG emissions might be unavoidable at the short-term, due to the unstable international food supply chains, mainly due to the current blockade that is affecting Qatar since 2017 by some surrounding countries of the Gulf Region and more recently to the COVID-19 crisis.

Conversely, the production of plant-based products would emit around one order of magnitude less GHGs than animal products. However, in order to keep the emissions under a relatively low level when producing regionally, vegetables production in Qatar should be done in an efficient way and by using lower- or non-CO_2_ emission technologies (e.g. renewable energies, precision and smart farming, re-use of organic by-products, use of treated wastewater…) and by implementing emerging food-system innovations like combining the production of plant-based products with fish farming (i.e. aquaponics systems). Considering the application of these technologies, and according to our results, a combination of regional and organic agriculture should be considered as a suitable GHG emission-mitigation option.

Therefore, we suggest a dietary change, which should be boosted by local authorities. According to our results, implementing the production and fostering the consumption of traditional organic animal products as well as the regional organic plant-based ones is highly recommendable in order to decrease the GHG emissions. In addition, implementing efficient and likewise sustainable innovations for indoor regional and organic agriculture should be prioritized. We encourage national authorities to include these recommendations in the Qatar National Food Security Strategy (2018–2023), not only on the supply-side, where some of them are already included, but also regarding the need for adapting diets to local conditions. In this regard, future studies should focus on the feasibility of coupling the increase in the food self-sufficiency of the country and the improvement in the sustainability of Qatar´s food system.

## Supplementary Information

**Additional file 1: Table S1.** Residue-to-product ratio (kg resiude Kg product^−1^), nitrogen content (Kg N Kg wet residue ^−1^ × 100), estimated direct N_2_O emissions (Kg CO_2_-eq Kg product^−1^) and reference of the residue-to-product ratio from the application of residues from harvest in organic farming. **Table S2.** Emission intensities from conventional and organic systems of cow milk (Kg CO_2_-eq Kg product^−1^) and conventional-to-organic ratio. **Table S3.** Emission intensities from conventional and organic systems of beef (Kg CO_2_-eq Kg product^−1^) and conventional-to-organic ratio. **Table S4.** Emission intensities from conventional and organic systems of poultry meat (Kg CO_2_-eq Kg product^−1^) and conventional-to-organic ratio. **Table S5.** Emission intensities from conventional and organic systems of pork (Kg CO_2_-eq Kg product-1) and conventional-to-organic ratio. **Table S6.** Emission intensities from conventional and organic systems of sheep and goat meat (Kg CO_2_-eq Kg product^−1^) and conventional-to-organic ratio. **Table S7**. Emission intensities from conventional and organic systems of farmed fish (Kg CO_2_-eq Kg product^−1^) and conventional-to-organic ratio. **Table S8.** Emission intensities from conventional and organic systems of feeding farmed salmon (Kg CO_2_-eq Kg product^−1^) and conventional-to-organic ratio. **Table S9.** Emission intensities from conventional and organic systems of eggs (Kg CO_2_-eq Kg product^−1^) and conventional-to-organic ratio. **Table S10.** Imports and local production of plant-based and animal products in Qatar in 2013 for each food commodity and for each type of product (in tones in the first two columns and in % in the last two columns) [26]. **Table S11.** Emissions per capita from transportation of imports for plant-based and animal products coming from the different countries of origin (Kg CO_2_ Kg capita^−1^). **Table S12.** Emissions from transportation of regional products (Kg CO_2_ Kg capita^−1^ and Kg CO_2_ kg product^−1^). **Table S13**. Summary of emissions from transportation of regional products and imports of plant-based and animal products (Kg CO_2_ kg product^−1^)

## Data Availability

The relevant results are shown in the manuscript, whereas other supporting data are shown in the Supplementary Material.

## Footnotes

1 Average value of estimated blue water required to produce different types of wheat, maize and barley [80]

2 Estimations from Brown [81] of 7 kg of grain per kilogram of beef and 2 kg per kilogram of poultry.

3 Estimated emissions of plant-based products are not a LCA, since emissions from tillage operations are not included in the calculations. However, according to our estimations, around 1547 kg CO_2_ ha^−1^ would be emitted from the use of inorganic fertilizers, whereas from tillage activities emissions would be between 7 and 56 kg CO_2_ ha^−1^ [102] and, thus, can be negligible for the overall calculation.
