# Supplementary material for: Can a shift to regional and organic diets reduce greenhouse gas emissions from the food system? A case study from Qatar
Source: Carbon Balance Manag. 2021 Jan 9;16:2. doi: 10.1186/s13021-020-00167-y (PMC7796384; doi:10.1186/s13021-020-00167-y)
Supplement: Supplementary file 1 — Additional file 1: Table S1. Residue-to-product ratio (kg resiude Kg product−1), nitrogen content (Kg N Kg wet residue −1 × 100), estimated direct N2O emissions (Kg CO2-eq Kg product−1) and reference of the residue-to-product ratio from the application of residues from harvest in organic farming. Table S2. Emission intensities from conventional and organic systems of cow milk (Kg CO2-eq Kg product−1) and conventional-to-organic ratio. Table S3. Emission intensities from conventional and organic systems of beef (Kg CO2-eq Kg product−1) and conventional-to-organic ratio. Table S4. Emission intensities from conventional and organic systems of poultry meat (Kg CO2-eq Kg product−1) and conventional-to-organic ratio. Table S5. Emission intensities from conventional and organic systems of pork (Kg CO2-eq Kg product-1) and conventional-to-organic ratio. Table S6. Emission intensities from conventional and organic systems of sheep and goat meat (Kg CO2-eq Kg product−1) and conventional-to-organic ratio. Table S7. Emission intensities from conventional and organic systems of farmed fish (Kg CO2-eq Kg product−1) and conventional-to-organic ratio. Table S8. Emission intensities from conventional and organic systems of feeding farmed salmon (Kg CO2-eq Kg product−1) and conventional-to-organic ratio. Table S9. Emission intensities from conventional and organic systems of eggs (Kg CO2-eq Kg product−1) and conventional-to-organic ratio. Table S10. Imports and local production of plant-based and animal products in Qatar in 2013 for each food commodity and for each type of product (in tones in the first two columns and in % in the last two columns) [26]. Table S11. Emissions per capita from transportation of imports for plant-based and animal products coming from the different countries of origin (Kg CO2 Kg capita−1). Table S12. Emissions from transportation of regional products (Kg CO2 Kg capita−1 and Kg CO2 kg product−1). Table S13. Summary of emissions from transportation of regio [file 13021_2020_167_MOESM1_ESM.docx]

**Table S1.** Residue-to-product ratio (kg resiude Kg product^-1^), nitrogen content (Kg N Kg wet residue ^-1^ x 100), estimated direct N_2_O emissions (Kg CO_2_-eq Kg product^-1^) and reference of the residue-to-product ratio from the application of residues from harvest in organic farming.

| **Crop** | **Residue:product ratio** | **N content** | **Estimated direct CO_2_-eq emissions** | **Source of residue:product ratio** |
| --- | --- | --- | --- | --- |
| Wheat and products | 1.28 | 0.700 | 0.053 | FAO [1] |
| Barley and products | 1.35 | 0.700 | 0.044 | FAO [1] |
| Maize and products | 1.96 | 0.690 | 0.063 | FAO [1] |
| Rye and products | 1.61 | 0.700 | 0.053 | FAO [1] |
| Oats | 1.42 | 0.700 | 0.047 | FAO [1] |
| Millet and products | 2.54 | 0.700 | 0.083 | FAO [1] |
| Cereals. Other | 1.64 | 0.700 | 0.054 | Average values from FAO [1] |
| Potatoes and products | 0.40 | 0.300 | 0.006 | Rosillo-Calle [2] |
| Sweet potatoes | 0.40 | 0.300 | 0.006 | Same as potatoes (assumption) |
| Tomatoes and products | 0.30 | 0.075 | 0.001 | Unal and Alibas [3] for Turkey (Assumption) |
| Onions | 1.20 | 0.105 | 0.006 | Williams *et al*. [4] |
| Vegetables. Other | 0.40 | 0.075 | 0.001 | Rosillo-Calle [2] |
| Sugar beet | 0.23 | 0.150 | 0.002 | Average value in EU-27 from Ecofys [5] |
| Pulses | 1.90 | 0.240 | 0.021 | Rosillo-Calle [2] |
| Grape | 0.61 | 0.740 | 0.021 | Unal and Alibas [3] for Turkey (Assumption) |
| Apples and products | 0.47 | 0.444 | 0.010 | Unal and Alibas [3] for Turkey (Assumption) |
| Olives | 1.37 | 0.930 | 0.060 | Unal and Alibas [3] for Turkey (Assumption) |
| Date Palm | 2.60 | 0.750 | 0.091 | FAO [1] |

**Table S2**. Emission intensities from conventional and organic systems of cow milk (Kg CO_2_-eq Kg product^-1^) and conventional-to-organic ratio.

| **Conventional** | **Organic** | **Conv/org** | **References** |
| --- | --- | --- | --- |
| 1.10 |  |  | Deittert *et al.* [6]. Grünberg *et al.* [7] |
| 1.05 |  |  | Schmidt and Osterburg [8], Grünberg *et al.* [7] |
| 0.85 | 0.78 | 1.09 | Hirschfeld *et al*.[9], Grünberg *et al.* [7] |
| 1.30 | 1.30 | 1.00 | Haas *et al.* [10] |
| 1.01 |  |  | LCA Food Database [11] |
| 1.06 | 1.23 | 0.86 | Williams *et al.* [4]) |
| 1.40 |  |  | Casey and Holden [12] |
| 1.40 | 1.50 | 0.93 | Thomassen *et al*. [13] |
| 0.90 | 0.94 | 0.96 | Cederberg and Flysjo [14] |
| 1.10 | 0.95 | 1.16 | Cederberg and Mattsson [15] |
| 1.09 |  |  | Phetteplace *et al.* [16] |
| 1.00 |  |  | Vergé *et al.* [17] |
| 0.72 |  |  | Basset-mens *et al.* [18] |
| 0.86 |  |  | Legard *et al.* [19] |
| 1.04 | 0.85 |  | Lindenthal *et al.* [20] |
| **1.06** | **1.06** | **1.00** |  |

**Table S3**. Emission intensities from conventional and organic systems of beef (Kg CO_2_-eq Kg product^-1^) and conventional-to-organic ratio.

| **Conventional** | **Organic** | **Conv/org** | **References** |
| --- | --- | --- | --- |
| 11.6 |  |  | LCA Food Database [11], Grünberg *et al.* [7] |
| 15.8 |  |  | Williams *et al*. [4], Grünberg *et al.* [7] |
| 25.3 |  |  | Williams *et al*. [4], Grünberg *et al.* [7] |
| 13.0 |  |  | Casey and Holden [12] |
| 14.8 |  |  | Subak [21] |
| 8.10 |  |  | Subak [21] |
| 6.04 | 3.93 | 1.54 | Hirschfeld *et al*.[9] |
| 14.54 | 12.25 | 1.19 | Hirschfeld *et al*.[9] |
| 8.40 | 13.5 | 0.62 | Hirschfeld *et al*.[9] |
| 16.76 | 16.28 | 1.03 | Hirschfeld *et al*.[9] |
| **13.43** | **12.28** | **1.09** |  |

**Table S4**. Emission intensities from conventional and organic systems of poultry meat (Kg CO_2_-eq Kg product^-1^) and conventional-to-organic ratio.

| **Conventional** | **Organic** | **Conv/org** | **References** |  |
| --- | --- | --- | --- | --- |
| **4.60** | **6.70** | **0.69** | Williams *et al*. [4] | |

**Table S5**. Emission intensities from conventional and organic systems of pork (Kg CO_2_-eq Kg product-1) and conventional-to-organic ratio.

| **Conventional** | **Organic** | **Conv/org** | **References** |
| --- | --- | --- | --- |
| 3.07 | 2.07 | 1.48 | Williams *et al*. [4] |
| 2.30 |  |  | LCA Food Database [11] |
| 6.40 | 5.60 | 1.14 | Williams *et al*. [4] |
| **3.92** | **2.99** | **1.31** |  |

**Table S6.** Emission intensities from conventional and organic systems of sheep and goat meat (Kg CO_2_-eq Kg product^-1^) and conventional-to-organic ratio.

| **Conventional** | **Organic** | **Conv/org** | **References** |
| --- | --- | --- | --- |
| **17.5** | **10.10** | **1.73** | Williams *et al*. [4] |

**Table S7**. Emission intensities from conventional and organic systems of farmed fish (Kg CO_2_-eq Kg product^-1^) and conventional-to-organic ratio.

| **Conventional** | **Organic** | **Conv/org** | **References** |
| --- | --- | --- | --- |
| 1.58 |  |  | Robb *et al.* [22]  Robb *et al.* [22]  Robb *et al.* [22] |
| 1.84 |  |  |  |
| 1.37 |  |  |  |
| 2.16 |  |  | Pelletier *et al.* [23] |
| 2.43 |  |  | Ziegler *et al.* [24] |
| 1.25 |  |  | Ziegler *et al.* [24] |
| **1.77** | **0.87** | **2.03*** |  |

* The emission intensity for the organic system has been calculated by applying the conv/org emission ratio from feeding farmed salmon (table S8)

**Table S8.** Emission intensities from conventional and organic systems of feeding farmed salmon (Kg CO_2_-eq Kg product^-1^) and conventional-to-organic ratio.

| **Conventional** | **Organic** | **Conv/org** | **Reference** |
| --- | --- | --- | --- |
| **1.4** | **0.69** | **2.03** | Pelletier and Tyedmers [25] |

**Table S9.** Emission intensities from conventional and organic systems of eggs (Kg CO_2_-eq Kg product^-1^) and conventional-to-organic ratio.

| **Conventional** | **Organic** | **Conv/org** | **References** |
| --- | --- | --- | --- |
| 5.58 | 7.05 | 0.79* | Williams *et al*. [4] |

* An average medium-size egg (49.61 g) has been selected for the assessment

**Table S10.** Imports and local production of plant-based and animal products in Qatar in 2013 for each food commodity and for each type of product (in tones in the first two columns and in % in the last two columns) [26].

| **Product** | **Available for consumption (ton)** | **Local consumption (ton)** | **Available for consumption from imports (%)** | **Local production (%)** |
| --- | --- | --- | --- | --- |
| Cereals | 265,395 | 2,084 |  |  |
| Fruits and dates | 140,451 | 32,988 |  |  |
| Vegetables | 388,025 | 43,446 |  |  |
| Total plant-based | 1,199,717 | 113,590 | **90.53** | **9.47** |
| Meat | 158,071 | 13,550 |  |  |
| Milk & dairy products products | 219,360 | 85,743 |  |  |
| Eggs | 28,292 | 4,365 |  |  |
| Fish | 38,853 | 12,005 |  |  |
| Total animal | 602,647 | 129,213 | **78.56** | **21.44** |

**Table S11.** Emissions per capita from transportation of imports for plant-based and animal products coming from the different countries of origin (Kg CO_2_ Kg capita^-1^). Percentage of imports and regionally produced commodities have been calculated using values from table S10. Emission factors for the different means of transport (Kg CO_2_ Kg product^-1^ and Km^-1^) are taken from table 4 in the manuscript and distances are shown in kilometers. Subtotal emissions for plant-based and animal products as well as total emissions are shown (Kg CO_2_ capita^-1^). Please, note that the total estimated emissions showed correspond to the BAU scenario (85% imports).

|  | **% imports** | | **Kg per capita imported** | | **Kg per capita local** | | **Mean of transport** | | **Emission factor** | | **Distance (Km)** | | **Kg CO_2_ per capita** | |
| --- | --- | --- | --- | --- | --- | --- | --- | --- | --- | --- | --- | --- | --- | --- |
|  | **Plant-based** | **Animal products** | **Plant-based** | **Animal** | **Plant-based** | **Animal** | **1** | **2** | **1** | **2** | **1** | **2** | **Plant-based** | **Animal** |
| United Arab Emirates | 16.42 | 8.06 | 48.75 | 11.63 | 5.10 | 3.17 | road |  | 0.000167 |  | 551 |  | 4.49 | 1.07 |
| Argentina | 1.91 |  | 5.67 |  | 0.59 |  | boat | plane | 0.000022 | 0.00112 | 15,911 | 13,321 | 43.31 | 0.00 |
| Australia | 4.85 | 18.43 | 14.39 | 26.59 | 1.51 | 7.26 | boat | plane | 0.000022 | 0.00112 | 13,303 | 12,371 | 101.82 | 79.91 |
| China | 2.51 |  | 7.46 |  | 0.78 |  | boat | plane | 0.000022 | 0.00112 | 10,825 | 6,763 | 29.13 | 0.00 |
| Jordan | 5.48 | 1.61 | 16.25 | 2.33 | 1.70 | 0.64 | road |  | 0.000167 |  | 2,029 |  | 5.51 | 0.79 |
| Egypt | 4.27 | 1.24 | 12.68 | 1.79 | 1.33 | 0.49 | road |  | 0.000167 |  | 2,646 |  | 5.60 | 0.79 |
| Spain | 2.43 |  | 7.20 |  | 0.75 |  | boat | plane | 0.000022 | 0.00112 | 8,775 | 5,073 | 21.16 | 0.00 |
| France | 4.01 | 1.62 | 11.89 | 2.33 | 1.24 | 0.64 | boat | plane | 0.000022 | 0.00112 | 8,484 | 4,645 | 32.04 | 2.78 |
| India | 24.95 | 4.22 | 74.07 | 6.09 | 7.75 | 1.66 | boat | plane | 0.000022 | 0.00112 | 2,408 | 2,294 | 97.12 | 3.39 |
| Italy | 1.77 |  | 5.25 |  | 0.55 |  | boat | plane | 0.000022 | 0.00112 | 7,917 | 4,043 | 12.34 | 0.00 |
| Lebanon | 2.24 |  | 6.66 |  | 0.70 |  | road |  | 0.000167 |  | 2,265 |  | 2.52 | 0.00 |
| Sri Lanka | 1.32 |  | 3.91 |  | 0.41 |  | boat | plane | 0.000022 | 0.00112 | 3,819 | 3,611 | 8.08 | 0.00 |
| Netherlands | 2.10 | 4.74 | 6.24 | 6.83 | 0.65 | 1.86 | boat | plane | 0.000022 | 0.00112 | 11,747 | 4,936 | 18.05 | 8.97 |
| Pakistan | 4.35 |  | 12.91 |  | 1.35 |  | boat | plane | 0.000022 | 0.00112 | 1,611 | 1,569 | 11.58 | 0.00 |
| Philippines | 2.79 |  | 8.28 |  | 0.87 |  | boat | plane | 0.000022 | 0.00112 | 9,166 | 7,292 | 34.63 | 0.00 |
| Russian Federation | 4.00 |  | 11.86 |  | 1.24 |  | plane |  | 0.00112 |  | 3,529 |  | 46.88 |  |
| Saudi Arabia | 5.59 | 25.31 | 16.58 | 36.52 | 1.73 | 9.97 | road |  | 0.000167 |  | 658 |  | 1.82 | 4.01 |
| Thailand | 1.57 |  | 4.67 |  | 0.49 |  | boat | plane | 0.000022 | 0.00112 | 8,221 | 5,262 | 14.19 | 0.00 |
| Turkey | 1.33 | 1.53 | 3.94 | 2.20 | 0.41 | 0.60 | boat | plane | 0.000022 | 0.00112 | 7,139 | 2,756 | 6.39 | 1.64 |
| Ukraine | 1.65 |  | 4.90 |  | 0.51 |  | plane | boat | 0.000022 | 0.00112 | 7,693 | 2,461 | 7.17 | 0.00 |
| United States | 4.47 | 3.99 | 13.26 | 5.75 | 1.39 | 1.57 | boat | plane | 0.000022 | 0.00112 | 15,164 | 10,789 | 82.35 | 15.44 |
| Belgium |  | 5.39 | 0.00 | 7.78 |  | 2.12 | boat | plane | 0.000022 | 0.00112 | 11,756 | 4,908 | 0.00 | 10.16 |
| Brazil |  | 13.96 | 0.00 | 20.14 |  | 5.50 | boat | plane | 0.000022 | 0.00112 | 15,109 | 11,526 | 0.00 | 57.35 |
| Denmark |  | 1.28 | 0.00 | 1.84 |  | 0.50 | boat | plane | 0.000022 | 0.00112 | 12,768 | 4,612 | 0.00 | 2.32 |
| United Kingdom |  | 3.94 | 0.00 | 5.69 |  | 1.55 | boat | plane | 0.000022 | 0.00112 | 11,671 | 5,247 | 0.00 | 7.85 |
| Kuwait |  | 1.42 | 0.00 | 2.05 |  | 0.56 | road |  | 0.000167 |  | 872 |  | 0.00 | 0.30 |
| New Zealand |  | 1.61 | 0.00 | 2.32 |  | 0.63 | boat | plane | 0.000022 | 0.00112 | 15,270 | 14,490 | 0.00 | 8.16 |
| Oman |  | 1.65 | 0.00 | 2.38 |  | 0.65 | road |  | 0.000167 |  | 992 |  | 0.00 | 0.39 |
| **Sub-total** |  |  | 296.85 | 144.26 | 31.05 | 39.37 |  |  |  |  |  |  | **586.17** | **205.31** |
| **Total** |  |  |  |  |  |  |  |  |  |  |  |  |  | **791.48** |

**Table S12.** Emissions from transportation of regional products (Kg CO_2_ Kg capita^-1^ and Kg CO_2_ kg product^-1^). Kilograms of local transportation have been estimated from tables S10 and S11. Emission factor (Kg CO_2_ Kg product^-1^ and Km^-1^) for transportation is taken from table 4 in the manuscript. An assumption of 75 Km as average distance for local transportation was considered.

| **Kg per capita local** | | **Mean of transport** | **Emission factor** | **Distance** | **Emissions per capita** | | **Emissions per unit of product** | |
| --- | --- | --- | --- | --- | --- | --- | --- | --- |
| **Plant-based** | **Animal** |  |  |  | **Plant-based** | **Animal** | **Plant-based** | **Animal** |
| 31.05 | 39.37 | Road (truck) | 0.000302 | 75 | 0.70318712 | 0.8917745 | 0.02265 | 0.02265 |

**Table S13**. Summary of emissions from transportation of regional products and imports of plant-based and animal products (Kg CO_2_ kg product^-1^)

|  | **Regional products** | | **Imported products** | |
| --- | --- | --- | --- | --- |
| **Emissions per unit of product** | **Plant-based** | **Animal** | **Plant-based** | **Animal** |
|  | 0.02265 | 0.02265 | 1.97 | 1.42 |

**References of the Supplementary Material**

1. FAO. Introduction How to use the BEFS Implementation Guide FAO ’ s Bioenergy and Food Security Approach The BEFS Approach Components BEFS Scoping Stakeholder Dialogue and Capacity Building Sustainable Bioenergy Assessment Support to Policy Formulation Risk Pre. 2018;

2. Rosillo-Calle F. Overview of Bioenergy. In: Rosillo-Calle F, de Groot P, Hemstock SL, Woods J, editors. The Biomass Assessment Handbook Bioenergy for a Sustainable Environment. London, Sterling, 1-26: Earthscan; 2007.

3. Unal H, Alibas K. Agricultural Residues as Biomass Energy. 2007;

4. Williams, A.G. Audsley, E. Sandars DL. Determining the environmental burdens and resource use in the production of agricultural and horticultural commoditites. Defra Research Project IS0205. 2006. p. 97.

5. Ecofys. The land use change impact of biofuels consumed in the EU: Quantification of area and The land use change impact of biofuels consumed in the EU. 2015.

6. Deittert C, Müller-Lindenlauf M, Athnmann M, Köpke U. Ökobilanz und Wirtschaftlichkeit ökologisch wirtschaftender Milchviehbetriebe mit unterschiedlicher Fütterungsintensität und Produktionsstruktur. 2008.

7. Grünberg J, Nieberg H, Schmidt T. Treibhausgasbilanzierung von Lebensmitteln ( Carbon Footprints ): Überblick und kritische Reflektion. Forestry. 2010;2010:53–72.

8. Schmidt TG, Osterburg B. Berichtsmodul “Landwirtschaft und Umwelt” in den umweltökonomischen Gesamtrechnungen: Projektbericht II - Methodische Erweiterungen. 2009.

9. Hirschfeld J, Preidl M, Korbun T. Klimawirkungen der Landwirtschaft in Deutschland. 2008.

10. Haas G, Wetterich F, Köpke U. Comparing intensive, extensified and organic grassland farming in southern Germany by process life cycle assessment. Agriculture, Ecosystems and Environment. 2001;83:43–53.

11. LCA Food Database. LCA Food Database [Internet]. Available from: www.lcafood.dk

12. Casey JW, Holden NM. Analysis of greenhouse gas emissions from the average Irish milk production system. Agricultural Systems. 2005;86:97–114.

13. Thomassen MA, van Calker KJ, Smits MCJ, Iepema GL, de Boer IJM. Life cycle assessment of conventional and organic milk production in the Netherlands. Agricultural Systems. 2008;96:95–107.

14. Cederberg C, Flysjo a. Life cycle inventory of 23 dairy farms in South-Western Sweden. SIK Rapport. 2004.

15. Cederberg C, Mattsson B. Life cycle assessment of milk production - a comparison of conventional and organic farming. Journal of Cleaner Production. 2000;8:49–60.

16. Phetteplace HW, Johnson DE, Seidl AF. Greenhouse gas emissions from simulated beef and dairy livestock systems in the United States. Nutrient Cycling in Agroecosystems. 2001;60:99–102.

17. Vergé XPC, Dyerm JA, Desjardins RL, Worth D. Greenhouse gas emissions from the Canadian dairy industry during 2001. Agricultural Systems. 2007;98:126–34.

18. Basset-mens C, Ledgard SF, Carran A. First life cycle assessment of milk production from New Zealand dairy farm systems. Conference, Palmerston North, New Zealand. 2005;2003:258–65.

19. Legard SF, Finlayson JD, Patterson MG, Carran RA, Wedderburn ME. Effects of intensification of dairy farming in New Zealand on whole-system resource use efficiency and environmental emissions. In: Halberg N, editor. Life cycle assessment in the agri-food sector. DIAS report, Animal Husbandry; 2004. p. 226–9.

20. Lindenthal T, Markut T, Rudolph G, Hanz K. Klimabilanz biologischer und konventioneller Lebensmittel im Vergleich. Ökologie & Landbau. 2010;2010.

21. Subak S. Global environmental costs of beef production. Ecological Economics. 1999;30:79–91.

22. Robb DHF, MacLeod M, Hasan MR, Soto D. Greenhouse gas emissions from aquaculture: a life cycle assessment from three Asian countries. Rome; 2017.

23. Pelletier N, Tyedmers P, Sonesson U, Scholz A, Ziegler F, Flysjo A, et al. Not all salmon are created equal: Life cycle assessment (LCA) of global salmon farming systems. Environmental Science and Technology. 2009;43:8730–6.

24. Ziegler F, Winther U, Hognes ES, Emanuelsson A, Sund V, Ellingsen H. The Carbon Footprint of Norwegian Seafood Products on the Global Seafood Market. Journal of Industrial Ecology. 2013;17:103–16.

25. Pelletier N, Tyedmers P. Feeding farmed salmon: Is organic better? Aquaculture. 2007;272:399–416.

26. Planning and Statistics Authority. Agricultural Statistics. 2013;
